# Overview and Methods for the Youth Risk Behavior Surveillance System — United States, 2023

**DOI:** 10.15585/mmwr.su7304a1

**Published:** 2024-10-10

**Authors:** Nancy D. Brener, Jonetta J. Mpofu, Kathleen H. Krause, Sherry Everett Jones, Jemekia E. Thornton, Zachary Myles, William A. Harris, David Chyen, Connie Lim, Loredona Arrey, Cecily K. Mbaka, Lindsay Trujillo, Shari L. Shanklin, Jennifer Smith-Grant, Lisa Whittle, Izraelle I. McKinnon, Malaika Washington, Barbara E. Queen, Alice M. Roberts

**Affiliations:** ^1^Division of Adolescent and School Health, National Center for Chronic Disease Prevention and Health Promotion, CDC, Atlanta, Georgia; ^2^U.S. Public Health Service Commissioned Corps, Rockville, Maryland; ^3^Epidemic Intelligence Service, CDC, Atlanta, Georgia; ^4^Westat, Rockville, Maryland; ^5^ICF International, Rockville, Maryland

## Abstract

The Youth Risk Behavior Surveillance System (YRBSS) is a set of surveys that tracks a broad range of behaviors, experiences, and conditions that can lead to poor health among high school students. The system includes a nationally representative Youth Risk Behavior Survey (YRBS) and separate school-based YRBSs conducted by states, tribes, territories, and local school districts. For the 2023 national YRBS, CDC made changes to the sampling method, survey administration mode, and questionnaire. Specifically, the sampling design added an American Indian or Alaska Native (AI/AN) supplemental sample so that separate, precise estimates could be made for AI/AN high school students, in addition to the usual sample designed to provide nationally representative data for the population of students in grades 9–12. To decrease the time needed to collect and process data, CDC changed the survey administration mode from paper-and-pencil scannable booklets to a tablet-based electronic survey. To provide national data on topics of emerging interest, CDC added new questions to the questionnaire. These new questions assessed social media use, experiences of racism at school, adverse childhood experiences, transgender identity, consent for sexual contact, and unfair discipline at school. Public health practitioners and researchers can use YRBSS data to examine the prevalence of youth health behaviors, experiences, and conditions; monitor trends; and guide interventions. This overview report describes 2023 YRBSS survey methodology, including sampling, data collection, data processing, weighting, and data analyses. The 2023 YRBS participation map, survey response rates, and a detailed examination of student demographic characteristics are included in this report. During 2023, in addition to the national YRBS, 68 site-level surveys were administered to high school students in 39 states, three tribal governments, five territories, and 21 local school districts. These site-level surveys use site-specific questionnaires that are similar to the national YRBS questionnaire but are modified to meet sites’ needs. This overview and methods report is one of 11 featured in this *MMWR* supplement, which reports results from the 2023 national YRBS but does not include data from the 68 site-level surveys. Each report is based on data collected using methods presented in this overview report. A full description of YRBSS results and downloadable data are available (https://www.cdc.gov/yrbs/index.html).

## Introduction

The Youth Risk Behavior Surveillance System (YRBSS) was established in 1991 to monitor priority health-risk behaviors among high school students in the United States. To meet changing needs, YRBSS has evolved to also monitor experiences and conditions affecting the health outcomes of this population. The system includes a national school-based survey administered by CDC and separate school-based surveys administered by states, tribal governments, territories, and local school districts (hereafter site-level or site surveys). These site-level surveys use site-specific questionnaires that allow modifications to the standard Youth Risk Behavior Survey (YRBS) questionnaire to meet state, tribal, territorial, and local needs. The standard YRBS questionnaire included 87 questions and is available at https://www.cdc.gov/healthyyouth/data/yrbs/pdf/2023/2023_YRBS_Standard_HS_Questionnaire.pdf. YRBS coordinators from participating states, tribes, territories, and local school districts voted for or against each proposed change, addition, and deletion. Final content of the standard YRBS questionnaire was decided based on the results of this voting process.

YRBSS offers a unique opportunity to monitor trends in risk behaviors, including some that have been monitored since 1991. As in previous cycles, the 2023 YRBSS measured student demographic characteristics (i.e., sex, sexual identity, race and ethnicity, age, and grade) and youth health behaviors, experiences, and conditions including those related to sexual activity, injury and violence, bullying, diet, physical activity, obesity, indicators of mental health, suicide-related behaviors, and substance use (i.e., electronic vapor product and tobacco product use, alcohol use, and other drug use).

For the 2023 YRBS, CDC made changes to the sampling method, survey administration mode, and questionnaire. The sampling design added an American Indian or Alaska Native (AI/AN) supplemental sample that maximized recruitment of AI/AN students. The purpose of this supplemental sample was to allow for separate, precise estimates to be made for AI/AN high school students; this was in addition to the usual sample designed to provide estimates for a nationally representative population of students in grades 9–12. To decrease the time needed to collect and process data, CDC changed the survey administration mode from paper-and-pencil scannable booklets to a tablet-based electronic survey. To provide national data on topics of emerging interest, CDC added new questions to the questionnaire. These new questions assessed social media use, experiences of racism at school, adverse childhood experiences, transgender identity, consent for sexual contact, and unfair discipline at school. Data from these new questions are highlighted in the reports in this *MMWR* supplement.

This report describes the 2023 YRBSS methodology, including sampling, data collection, data processing, weighting, and data analyses. This overview and methods report is one of 11 reports in the *MMWR* supplement featuring 2023 YRBS data. The other 10 reports provide the most recent national data on the following topics: 1) health behaviors and experiences among AI/AN students; 2) social media use; 3) experiences of racism at school; 4) adverse childhood experiences (ACEs); 5) mental health and suicidal thoughts and behaviors; 6) transgender identity; 7) asking for consent, verbally, at last sexual contact; 8) breakfast consumption; 9) physical activity; and 10) report of unfair discipline at school. In total, five individual questions and one set of eight questions (ACEs) were added to the 2023 YRBS questionnaire to examine urgent and emerging student health behaviors and experiences. Along with results from site-level surveys, public health practitioners and researchers can use YRBS data to examine the prevalence of youth health behaviors, experiences, and conditions; monitor trends; and guide interventions. This supplement does not include data from site-level surveys; however, those results can be found in CDC’s web-based applications for YRBSS data, including YRBS Explorer (https://yrbs-explorer.services.cdc.gov), Youth Online (https://nccd.cdc.gov/youthonline/App/Default.aspx), and the YRBS Analysis Tool (https://yrbs-analysis.cdc.gov).

## National YRBS Methodology

### Overview

Historically, YRBS has been administered during the spring of odd-numbered years to students in grades 9–12 enrolled in U.S. public and private schools. Although the previous YRBS was not administered until fall 2021 because of the COVID-19 pandemic, the 2023 survey resumed the typical timing and was conducted during the spring semester (January–June) 2023. Biennial administration of the YRBS allows CDC to assess temporal changes in behaviors among the U.S. high school population. YRBS, conducted among a nationally representative sample of students in grades 9–12 enrolled in U.S. public and private schools, provides comparable data across survey years and allows for comparisons between national and site-level data.

### Questionnaire

The YRBS questionnaire uses single-item measures to monitor and describe a wide variety of health behaviors and conditions. In 2023, the questionnaire consisted of 107 questions. Of those, 87 questions were included in the standard questionnaire all sites used as the basis for their site-level questionnaires. Twenty questions were added to the standard questionnaire that reflected areas of particular interest for CDC and other partners. As in all cycles, the previous year’s standard questionnaire was revised to allow for the inclusion of questions assessing emerging issues and risk behaviors among high school students. Subject matter experts from CDC, academia, other Federal agencies, and nongovernmental organizations proposed changes, additions, and deletions to the questionnaire. CDC made further refinements to the questionnaire on the basis of feedback from cognitive testing with high school students. The YRBS questionnaire was offered in both English and Spanish.

All questions, except those assessing height, weight, and race, were multiple choice, with a maximum of eight mutually exclusive response options and only one possible answer per question. A recent test-retest study of most of the 2023 survey questions demonstrated substantial reliability among these questions (*1*). The wording of each question, including recall periods, response options, and operational definitions for each variable, are available in the 2023 YRBS questionnaire and data user’s guide. (YRBSS data and documentation are available at https://www.cdc.gov/yrbs/data/index.html.)

The shift from paper-and-pencil to electronic survey administration allowed CDC to introduce new questionnaire features. First, for questions related to tobacco products, prescription opioid medicine, and contraceptives, the tablet displayed images to enhance students’ understanding of the question or response options. Second, the questionnaire included skip patterns, meaning that students who responded that they did not engage in a particular behavior (e.g., current cigarette smoking) were not shown subsequent questions regarding that behavior (e.g., number of cigarettes smoked per day). Questions that were skipped appropriately based on responses to a previous question were not coded as missing in the data set, but instead with a response option noting that the student did not engage in the behavior measured in the subsequent question. For example, a student who responded “no” to “Have you ever smoked a cigarette, even one or two puffs?” would not be shown the question, “During the past 30 days, on how many days did you smoke cigarettes?” but their response to that question in the data set would be coded as 0 days. Third, electronic data collection allowed for real-time logic checks, reducing the amount of editing required after data collection (i.e., the questionnaire was programmed so that if students entered an invalid response for items such as height and weight, they were prompted to correct it).

### Sampling

The sample for the 2023 YRBS included two components. The main sample was designed to provide nationally representative data. The supplemental sample was designed to be used in combination with the main sample to increase the number of AI/AN participants.

#### Main Sample

For the main sample, the sampling frame consisted of all regular public schools (including charter schools), parochial schools, and other private schools with students in at least one of grades 9–12 in the 50 U.S. states and the District of Columbia. Alternative schools, special education schools, schools operated by the U.S. Department of Defense or the Bureau of Indian Education, and vocational schools serving students who also attended another school were excluded. Schools with ≤40 students enrolled in grades 9–12 (combined) also were excluded. The sampling frame was constructed from data files obtained from MDR (formerly Market Data Retrieval) and the National Center for Education Statistics (NCES). NCES data sources included the Common Core of Data (https://nces.ed.gov/ccd) for public schools and the Private School Survey (https://nces.ed.gov/surveys/pss) for private schools.

A three-stage cluster sampling design was used to produce a nationally representative sample of students in grades 9–12 who attend public and private schools. The first-stage sampling frame comprised 1,257 primary sampling units (PSUs), which consisted of entire counties, groups of smaller adjacent counties, or parts of larger counties. PSUs were categorized into 16 strata according to their metropolitan statistical area status (i.e., urban or nonurban) and the percentages of Black or African American (Black) and Hispanic or Latino (Hispanic) students in each PSU. Of the 1,257 PSUs, 60 were sampled with probability proportional to overall school enrollment size for that PSU. For the second-stage sampling, secondary sampling units (SSUs) were defined as a physical school with grades 9–12 or a school created by combining nearby schools to provide all four grades. From the 60 PSUs, 180 SSUs were sampled with probability proportional to school enrollment size. To provide adequate coverage of students in small schools, an additional 20 small SSUs were selected from a subsample of 20 of the 60 PSUs. These 200 SSUs corresponded to 204 physical schools. The third stage of sampling comprised random sampling of one or two classrooms in each of grades 9–12 from either a required subject (e.g., English or social studies) or a required period (e.g., homeroom or second period). All students in sampled classes who could independently complete the questionnaire were eligible to participate. Schools, classes, and students that refused to participate were not replaced.

#### Supplemental Sample

The sampling frame for the AI/AN supplemental sample was constructed using the same data sources and process used for the main sampling frame. As an additional step, the sampling frame was restricted to public schools with an estimated enrollment of ≥28 students in each grade to most efficiently reach AI/AN students. As with the main sample, Bureau of Indian Education schools were not included in the frame because of their unique nature and location on lands that often are tribally controlled (*2*). Although this more restricted frame limited the coverage when using the supplemental sample alone, sample representation of the AI/AN population was expanded when the supplemental sample was combined with the main sample, which represents all schools, including schools with <28 students in each grade as well as nonpublic schools.

As with the main sample, the supplemental sample used a three-stage cluster sampling design. The first-stage sampling frame comprised the same 1,257 PSUs, of which 55 SSUs were sampled with probability proportional to the aggregate AI/AN school enrollment size in grades 9–12. These 55 SSUs corresponded to 114 physical schools. The third stage of sampling followed the same process as for the main sample, except that two classrooms in each grade were selected to participate to maximize the number of AI/AN students.

### Data Collection Procedures

Institutional review boards at CDC and ICF, the survey contractor, approved the protocol for YRBS. Data collection was conducted consistent with applicable Federal law and CDC policy.* Survey procedures were designed to protect students’ privacy by allowing for anonymous participation. Participation was voluntary, and local parental permission procedures were followed before survey administration. During survey administration, students completed the self-administered questionnaire during one class period using tablets that had been programmed with the survey instrument. Trained data collectors visited each school to distribute the tablets to the students and collect them after survey completion. The tablets were not connected to the Internet. Instead, students’ data were saved to the tablets, and data collectors synchronized all locally stored data to a central repository at the end of each day.

The shift from paper-and-pencil to electronic questionnaire administration provided several benefits. First, electronic data collection reduced the time needed for students to complete the survey. Whereas the paper-and-pencil version of the survey used in previous cycles took a full 45-minute class period to complete, the tablet version was typically completed in 25 minutes. This decrease is a result of the increased speed of touching a response on a tablet compared with filling a bubble on a scannable booklet using a pencil, as well as the use of skip patterns. Further, students have been found to prefer electronic surveys over paper-and-pencil surveys because of their familiarity with and comfort using electronic devices (*3*). Third, electronic administration eliminated the use of paper. Not only is this a more environmentally friendly approach, but it also increased the speed at which the data could be compiled. Rather than waiting for completed booklets to be shipped and scanned, data were available for processing as soon as the tablets were synchronized. This also allowed CDC to track data collection progress in nearly real-time. Finally, students who were absent on the day of data collection and could not complete the questionnaire on a tablet were able to complete a web-based version of the questionnaire in a setting similar to the tablet administration when they returned to school; 323 surveys were completed using this web-based platform rather than the tablet, which increased overall completion rates by eliminating the need for schools to mail questionnaires back to the survey contractor.

### Response Rates and Data Processing

The main sample and the AI/AN supplemental sample were combined to create a single sample file for the 2023 national survey. At the end of the data collection period, 20,386 questionnaires were completed in 155 schools. The national data set was cleaned and edited for inconsistencies. Missing data were not statistically imputed. A questionnaire failed quality control when <20 responses remained after editing or when it contained the same answer to ≥15 consecutive questions. Among the 20,386 completed questionnaires, 283 failed quality control and were excluded from analysis, resulting in 20,103 usable questionnaires. The school response rate was 49.8%, the student response rate was 71.0%, and the overall response rate (i.e., [student response rate] x [school response rate]) was 35.4%.

Race and ethnicity were ascertained from two questions: 1) “Are you Hispanic or Latino?” (yes or no) and 2) “What is your race?” (American Indian or Alaska Native [AI/AN], Asian, Black or African American [Black], Native Hawaiian or other Pacific Islander [NH/OPI], or White). For the second question, students could select more than one response option. (Persons of Hispanic or Latino origin might be of any race but are categorized as Hispanic; all racial groups are non-Hispanic.) Except for the report in this *MMWR* supplement that focused on AI/AN students, students were classified as Hispanic or Latino and are referred to as Hispanic if they answered “yes” to the first question, regardless of how they answered the second question. For example, students who answered “no” to the first question and selected only Black or African American to the second question were classified as Black or African American and are referred to as Black. Likewise, students who answered “no” to the first question and selected only White to the second question were classified and are referred to as White. Race and ethnicity were classified as missing for students who did not answer the first question and for students who answered “no” to the first question and did not answer the second question. Students who selected more than one response option to “What is your race?” were classified as multiracial. This classification of race and ethnicity aligns with the Office of Management and Budget standards in place at the time of the survey (https://www.govinfo.gov/content/pkg/FR-1997-10-30/pdf/97-28653.pdf). Although using uniform classifications facilitates trend interpretation and between-group comparisons, preferred terminology classification practices are evolving; the Office of Management and Budget released new standards after the 2023 YRBS cycle was completed (https://www.federalregister.gov/documents/2024/03/29/2024-06469/revisions-to-ombs-statistical-policy-directive-no-15-standards-for-maintaining-collecting-and). In addition, the unilateral classification of race and ethnicity does not describe the heterogeneity and unique experiences of students within a particular racial or ethnic group (*4*).

To obtain a sufficient sample size for analyses of health behaviors, experiences, and conditions by sexual identity, students were categorized as heterosexual if they chose that response option, and students who responded as gay or lesbian, bisexual, “I describe my sexual identity some other way,” or “I am not sure about my sexual identity/questioning” were usually grouped together as LGBQ+ (Table 1). Although this binary categorization often was necessary for statistical analysis, LGBQ+ populations are not a single homogeneous group, and this categorization might result in a loss of understanding the unique experiences of these sexual identity subgroups (*5*). Students also were categorized into those who had no sexual contact, those who had sexual contact with only the opposite sex, or those who had sexual contact with only the same sex or with both sexes on the basis of their responses to the question, “During your life, with whom have you had sexual contact?” Students who had no sexual contact were excluded from analyses related to sexual behaviors. Female students who had sexual contact with only females were excluded from analyses on condom use.

**TABLE 1:** Questions, response options, and analytic coding for sexual identity and sexual contacts — Youth Risk Behavior Survey, United States, 2023

Question	Response option		Analytic coding
**Sexual identity**			
Which of the following best describes you? 1) Heterosexual (straight), 2) gay or lesbian, 3) bisexual, 4) I describe my sexual identity some other way, 5) I am not sure about my sexual identity/questioning, or 6) I do not know what this question is asking	Heterosexual (straight) (1), gay or lesbian (2) or bisexual (3), describe identity some other way (4), questioning (5), or did not understand (6)		Heterosexual students (1); lesbian, gay, or bisexual students (2 or 3); students who describe identity in some other way (4); questioning students (5); or students missing sexual identity variable (6)
**Sex of sexual contacts**			
During your life, with whom have you had sexual contact? 1) I have never had sexual contact, 2) females, 3) males, or 4) females and males What is your sex? 1) Female or 2) male	I have never had sexual contact*		Students who had no sexual contact
	**Contact:** Female Male	**Student:** Male Female	Students who had sexual contact with only the opposite sex
	**Contact:** Male Females and males Female Females and males	**Student:** Male Male Female^†^ Female	Students who had sexual contact with only the same sex or with both sexes

* Excluded from analyses on sexual behaviors.

^†^ Excluded from analyses on condom use.

### Weighting

Weights were applied to the final sample so that responses were generalizable to the U.S. student population in grades 9–12. For the 2023 YRBS, weights were calculated separately for the main sample and the AI/AN supplemental sample. The calculation of the weights followed the same process for both samples. First, a weight was applied based on student sex, race and ethnicity, and grade to each record to adjust for school and student nonresponse. Next, the two weighted data sets were concatenated and combined weights were calculated as final survey weights. Finally, the overall weights were scaled so that the weighted count of students equaled the total sample size, and the weighted proportions of students in each grade matched the national population proportions. Therefore, in the national data set, weighted estimates are nationally representative of all students in grades 9–12 attending U.S. public and nonpublic schools.

### Analytic Methods

Findings presented in this *MMWR* supplement are derived from analytic procedures similar to what is described in this overview report. For more information about the detailed analyses presented in other reports in this supplement (e.g., variables analyzed, custom measures, and data years), see Methods in each individual report.

All statistical analyses used SAS-callable SUDAAN (version 11.0.3 or 11.0.4; RTI International) to account for the complex sampling design and weighting. In all reports, prevalence estimates and CIs were computed for variables used in those reports. Prevalence estimates where the denominator was <30 were considered statistically unreliable and therefore were suppressed. In certain reports, chi-square tests were used to examine associations between health behaviors, experiences, or conditions and demographic characteristics (e.g., sex, race and ethnicity, grade, sexual identity, and sex of sexual contacts). Pairwise differences between groups (e.g., male versus female students) were determined using *t*-tests. All analyses used a domain analysis approach to make certain the accurate calculation of standard errors, CIs, and p values despite missing data in certain variables. Prevalence differences and ratios were calculated using logistic regression with predicted marginals. All prevalence estimates and measures of association used Taylor series linearization. All tests were considered statistically significant at the p<0.05 level. Prevalence ratios were considered statistically significant if 95% CIs did not cross the null value of 1.0.

For analyses of temporal trends reported in the YRBSS web applications and the Youth Risk Behavior Survey Data Summary & Trends Report: 2013–2023 (https://www.cdc.gov/yrbs/dstr/index.html), logistic regression analyses were used to examine linear and quadratic changes in estimates, controlling for sex, grade, and racial and ethnic changes over time. A p value of <0.05 associated with a regression coefficient was considered statistically significant. Linear and quadratic time variables were treated as continuous and were coded using orthogonal coefficients calculated with PROC IML in SAS (version 9.4; SAS Institute). A minimum of 3 survey years was required for calculating linear trends, and a minimum of 6 survey years was required to calculate quadratic trends. Separate regression models were used to assess linear and quadratic trends. When a significant quadratic trend was identified, Joinpoint (version 5.02; National Cancer Institute) was used to automate identification of the year when the trend changed. Then, regression models were used to identify linear trends occurring before and after the change in trend. A quadratic trend indicates a statistically significant but nonlinear change in prevalence over time. A long-term temporal change that includes a significant linear and quadratic trend demonstrates nonlinear variation (e.g., leveling off or change in direction) in addition to an overall increase or decrease over time. Cubic and higher-order trends were not assessed.

For analyses of 2-year changes in the YRBSS web applications, prevalence estimates from 2021 and 2023 were compared by using *t*-tests for behaviors, experiences, or conditions assessed with identically worded questions in both survey years. Prevalence estimates were considered statistically different if the *t*-test p value was <0.05.

### Data Availability and Dissemination

National and site-level YRBS data (1991–2023) are available in a combined data set from the YRBSS data and documentation website (https://www.cdc.gov/yrbs/data/index.html), as are additional resources, including data documentation and analysis guides. Data are available in both Access and ASCII formats, and SAS and SPSS programs are provided for converting the ASCII data into SAS and SPSS data sets. Variables are standardized to facilitate trend analyses and for combining data. YRBSS data also are available online via three web-based data dissemination tools: Youth Online, YRBS Analysis Tool, and YRBS Explorer. Youth Online allows point-and-click data analysis and creation of customized tables, graphs, maps, and fact sheets (https://nccd.cdc.gov/Youthonline/App/Default.aspx). Youth Online also performs statistical tests by health topic and filters and sorts data by race and ethnicity, sex, grade, and sexual orientation. The YRBS Analysis Tool allows real-time data analysis of YRBS data that generates frequencies, cross-tabulations, and stratified results (https://yrbs-analysis.cdc.gov). YRBS Explorer is an application featuring options to view and compare national, state, and local data via tables and graphs (https://yrbs-explorer.services.cdc.gov).

## State, Tribal, Territorial, and Local School District YRBS Methodology

### Overview

**FIGURE 1:**
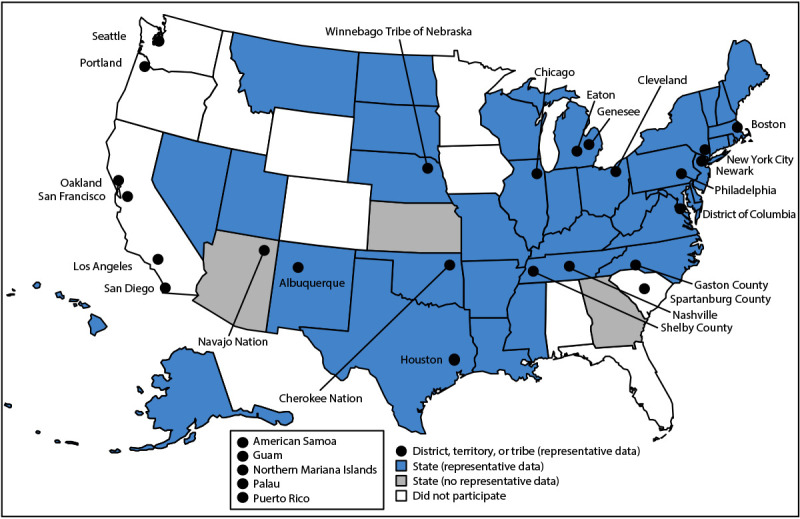
State, tribal government, territorial, and local school district Youth Risk Behavior Surveys — selected U.S. sites, 2023

**FIGURE 2:**
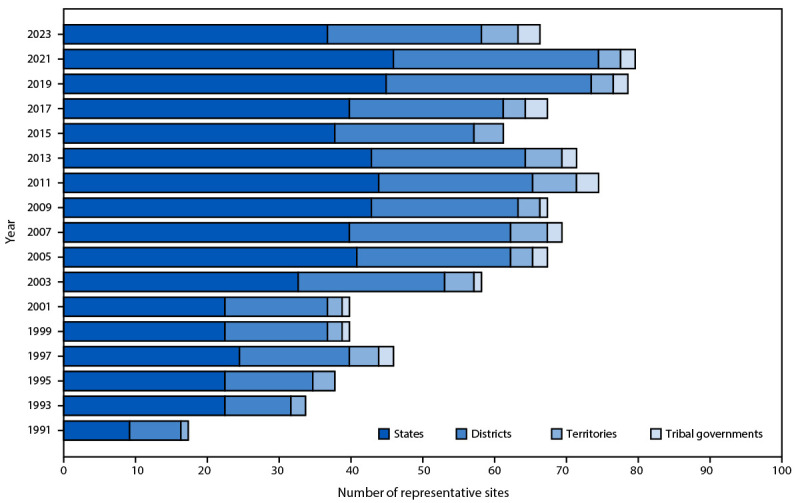
Number of states, local school districts, territories, and tribal governments with representative Youth Risk Behavior Survey data, by year of survey — selected U.S. sites, Youth Risk Behavior Survey, 1991–2023

Biennial administration of site-level YRBSs allows state, tribal, territorial, and local education and health agencies to monitor health behaviors, experiences, and conditions among the high school populations in their respective jurisdictions. Site-level survey data provide comparable data across years within jurisdictions and allow for comparisons of data across jurisdictions (e.g., national to state). Site-level surveys are conducted among students in grades 9–12 attending public schools using samples representative of their jurisdiction. Sixty-eight sites conducted a YRBS in 2023 (39 states, three tribal governments, five territories, and 21 local school districts) (Figures 1 and 2). Four sites administered their surveys during fall 2022, 45 during spring 2023, and 19 during fall 2023. The survey is self-administered anonymously and takes one class period (approximately 45 minutes) or less to complete. Each jurisdiction followed requirements for institutional review board approval of the survey protocols for their respective YRBSs. Survey methodology for data collection, processing, and analytic methods were the same as those described for the national YRBS; however, 32 sites collected data electronically using computers, smartphones, or tablets, and 36 collected data using paper-and-pencil questionnaires and scannable answer sheets.

### Questionnaires

The 2023 YRBS standard questionnaire contained 87 questions and was used as the starting point for site-level YRBS questionnaires. Sites could add or delete questions but were required to use at least 58 of the questions on the standard questionnaire, including all demographic questions. This flexibility allowed YRBS coordinators and other state and local partners the opportunity to include topics of interest by customizing their survey.

### Sampling

Sites used a two-stage cluster sampling design to produce a representative sample of students in grades 9–12 in their jurisdictions. In 37 states, one tribe, one territory, and four local school districts, in the first sampling stage, public schools with any of grades 9–12 were sampled with probability proportional to school enrollment size. In two states, two tribes, four territories, and 17 local school districts, all schools in the jurisdiction were selected to participate (i.e., a census of schools). In the second sampling stage, classes from either a required subject (e.g., English or social studies) or a required period (e.g., homeroom or second period) were sampled randomly. In seven sites (Vermont, District of Columbia, Navajo Nation, Winnebago Tribe of Nebraska, American Samoa, Northern Mariana Islands, and Palau), a census of students was selected to participate. All students in selected classes who could independently complete the survey were eligible to participate.

Students in schools that were in both the national sample and a site-level sample were asked to participate in only one YRBS. Either the national questionnaire or the site’s questionnaire was administered to the students in these schools, and data from questions included on both questionnaires were shared between sites. Because the site questionnaires differ from the national questionnaire, students in these schools were not asked all national YRBS questions. Therefore, for national data, the total number of students answering each question varied.

### Response Rates, Nonresponse Bias Analyses, and Weighting

Site-level data sets were cleaned and edited for inconsistencies. Missing data were not statistically imputed. A questionnaire failed quality control when <20 responses remained after editing or when it contained the same answer to ≥15 consecutive questions. CDC conducted nonresponse bias analyses for all sites to determine whether data for each site could be weighted to be representative of its jurisdiction. These analyses compared responding and nonresponding schools on school enrollment size (small, medium, or large), a measure of the school’s poverty level (usually the percentage of students eligible for free or reduced-price lunch), and locale type (city, suburban, town, or rural). Analyses also compared responding and nonresponding students by grade and weighted sample and population percentages by grade, sex, and race and ethnicity. If limited statistically significant differences between comparison groups were found, data were weighted to be representative of their respective populations.

A weight calculated as the product of school base weight, student base weight, school nonresponse adjustment factor, student nonresponse adjustment factor, and poststratification adjustment factor was based on student sex, grade, and race and ethnicity and attached to each record to adjust for school and student nonresponse in each jurisdiction. The weighted count of students equals the student population in each jurisdiction. A total of 36 states, three tribal governments, five territories, and 21 local school districts had representative (weighted) data in 2023 (Figures 1 and 2). In 15 states and 13 local school districts, weighted estimates were representative of all students in grades 9–12 attending regular public schools, and in 21 states and eight local school districts, weighted estimates were representative of regular public-school students plus students in grades 9–12 in other types of public schools (e.g., alternative or vocational schools).

### Data Availability and Dissemination

A combined data set including national, state, and local school district YRBS data (1991–2023) is available from the YRBSS data and documentation website (https://www.cdc.gov/yrbs/data/index.html). Availability of site data depends on survey participation, data quality, and data-sharing policies. Information about YRBSS data is available on the participation maps and history website (https://www.cdc.gov/yrbs/data/yrbs-participation.html). Site-level YRBS data collected during 1991–2023 are available through Youth Online (https://nccd.cdc.gov/Youthonline/App/Default.aspx), the YRBS Analysis Tool (https://yrbs-analysis.cdc.gov), and YRBS Explorer (https://yrbs-explorer.services.cdc.gov).

## Response Rates and Nonresponse Bias Analyses

**FIGURE 3:**
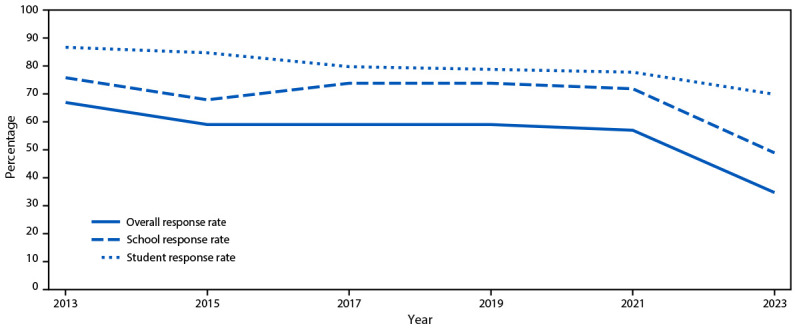
Overall, school, and student response rates for the Youth Risk Behavior Survey — United States, 2013–2023

The 2023 YRBS overall response rate of 35.4% was the lowest in the history of the survey. Although the student response rate of 71.0% was only slightly lower than in previous cycles (Figure 3), the school response rate of 49.8% was substantially lower. Overall response rates in YRBS have decreased steadily since 2011; these rates have been in the low 60% range since the 2015 cycle and <60% post-COVID in 2021. However, research indicates that a high survey response rate does not necessarily result in an unbiased sample, and that nonresponse bias is not necessarily lower in samples with a higher response rate compared with those with a lower response rate (*6*). For the YRBS, nonresponse bias analyses included bivariate and multivariate analyses of school and student-level characteristics associated with nonresponse. Bivariate analyses revealed significant differences between participating and nonparticipating schools in school type, locale, percentage of Asian students, current per-pupil expenditures, and whether the school offers career and technical education. In a multivariate logistic regression model that included all these variables, only per-pupil expenditure remained a significant predictor of school participation; schools with lower per-pupil expenditure were less likely to participate. Weighting adjustments accounted for nonresponding schools and minimized nonresponse bias (ICF, unpublished data, 2024).

## Demographic Characteristics

The 2023 YRBS data were weighted to match national population proportions. After weighting, approximately half of students were male (51.9%), and percentages of students by grade were as follows: grade 9 (26.4%), grade 10 (25.8%), grade 11 (24.2%), and grade 12 (23.3%) (Table 2). In addition, 48.1% of students were White, followed by Hispanic (27.4%), Black (13.3%), multiracial (6.1%), Asian (4.3%), NH/OPI (0.4%), and AI/AN (0.3%).

**TABLE 2:** Student demographic characteristics — Youth Risk Behavior Survey, United States, 2023

Characteristic	No. (%)
Student sample size*	**20,103 (100)**
**Sex^†^**	
Female	9,884 (48.1)
Male	10,061 (51.9)
**Race and ethnicity^§,¶^**	
American Indian or Alaska Native	1,334 (0.3)
Asian	995 (4.3)
Black or African American	1,791 (13.3)
Native Hawaiian or other Pacific Islander	105 (0.4)
White	9,700 (48.1)
Hispanic or Latino	3,994 (27.4)
Multiracial	1,814 (6.1)
Grade**	
9	5,680 (26.4)
10	5,410 (25.8)
11	4,811 (24.2)
12	3,961 (23.3)
**Sexual identity^††^**	
Heterosexual (straight)	13,289 (73.3)
Gay or lesbian	683 (4.0)
Bisexual	2,053 (11.4)
Describe sexual identity in some other way	760 (4.3)
Not sure about sexual identity/questioning	850 (4.4)

* Among the 20,386 completed questionnaires, 283 failed quality control and were excluded from analysis, resulting in 20,103 usable questionnaires.

^†^ Does not include 158 students who did not indicate sex.

^§^ Persons of Hispanic or Latino origin might be of any race but are categorized as Hispanic; all other racial groups are non-Hispanic.

^¶^ Does not include 370 students who did not indicate race, ethnicity, or both.

** Does not include 48 students who responded, “ungraded or other grade” and 193 students who did not indicate a grade.

^††^ Does not include 465 students who responded, “I do not know what this question is asking” and 2,003 students with missing data.

In 2023, 73.3% of students self-identified as heterosexual, 4.0% as gay or lesbian, 11.4% as bisexual, and 4.4% as questioning; 4.3% responded with “I describe my sexual identity some other way,” and 2.5% responded with “I do not know what this question is asking” (Table 2). In 2023, a total of 53.7% of students reported no sexual contact during their lives. An estimated 38.1% of students had sexual contact with the opposite sex only, 5.1% with both sexes, and 3.0% with the same sex only.

## Discussion

The 2023 YRBS implemented multiple features that improved the quality and usability of the data. Specifically, the transition from paper-and-pencil to electronic administration helps align YRBS to CDC’s Data Modernization Initiative, and decreases the time needed for data collection and processing. Importantly, previous studies demonstrated that electronic administration did not affect prevalence estimates (*7*,*8*). The addition of the AI/AN supplemental sample provides improved precision of nationwide estimates of AI/AN students’ health behaviors, experiences, and conditions for the first time in the history of YRBSS. Such data are critical to developing interventions that address the unique needs of AI/AN students. The addition of ACEs questions provides the first nationally representative adolescent data on these experiences, which can also guide interventions developed to prevent and mitigate the effects of ACEs (*9*).

In 2023, overall response rates for the YRBS fell below 40%, continuing a previously reported decline (*10*). These numbers reflect the challenges of obtaining approvals for survey participation at both the school district and school levels. Disinformation campaigns targeting YRBSs across the country also contribute to declining YRBS response rates (*11*). Such campaigns misrepresent survey content, data collection procedures, and data utility. YRBSS continues to collect high quality data via working with state and local partners, using a rigorous complex sample design to ensure that sampled schools are representative of high schools in the United States, oversampling Black and Hispanic students so that estimates derived from their responses are precise, and using weights to adjust for and minimize nonresponse bias and conducting thorough nonresponse bias analyses.

New questions featured in the 2023 YRBS expand on the reach of youth health data and address important issues affecting youths. For example, the report focused on AI/AN students used an inclusive approach to coding race and ethnicity such that all AI/AN students, even those who also identified as another race or as Hispanic, were included as AI/AN. Among AI/AN students, the protective factors of household adult caretaking, parental monitoring, and school connectedness were associated with lower prevalence of substance use, mental health problems and suicide risk, and experiences with violence (*12*). Findings from the report on social media use indicate that approximately three fourths of students reported using social media at least several times a day. This level of social media use was associated with a higher prevalence of bullying victimization at school and electronically, persistent feelings of sadness or hopelessness, seriously considering attempting suicide, and making a suicide plan (*13*). The report about racism found that approximately one in three students had ever experienced racism at school, with higher estimates for Asian, multiracial, and Black students. Students who experienced racism had a higher prevalence of health risk behaviors and experiences. These findings demonstrate that racism is experienced by students within the school setting and continues to disproportionately affect adolescents in racial and ethnic groups that have been marginalized (*14*). The report on ACEs found that ACEs were common, with 76.1% of adolescents reporting ≥1 ACE and 18.5% experiencing ≥4 ACEs. Adolescents who experienced ≥4 ACEs were more likely to identify as female, AI/AN, multiracial, gay or lesbian, bisexual, or to describe their sexual identity in some other way (*9*). The report on transgender identity established that, nationally, 3.3% of adolescents identify as transgender and an additional 2.2% are questioning whether they identify as transgender. Transgender and questioning adolescents have a higher prevalence of experiencing violence, poor mental health, suicidal thoughts and behaviors, and unstable housing and a lower prevalence of school connectedness compared with their cisgender peers (*15*). The report about sexual consent found that 79.8% of high school students asked for consent verbally at last sexual contact. In addition, students who asked for sexual consent verbally were less likely to report first sexual intercourse before age 13 and were more likely to use condoms (*16*). Findings for the report about unfair discipline at school indicated that 19.1% of students reported experiencing unfair discipline during the past year. Black students (23.1%) had a higher prevalence of reporting unfair discipline compared with White students (18.1%). Furthermore, students who reported receiving unfair discipline were more likely to engage in various health risk behaviors (e.g., skipping school because of feeling unsafe, carrying a weapon on school property, and attempting suicide). These findings highlight the significance of addressing school discipline as a public health issue and intervening on the underlying social drivers of inequity in disciplinary action (*17*). Taken together, these findings from the new questions in the 2023 YRBS document important challenges that adolescents face and, in uncovering these realities, public health practitioners, schools, and families can use these data to take action.

In addition to new questions, prevalences and patterns in health behaviors identified in other reports on longstanding YRBS topics also reinforced the need for specific, tailored public health interventions and resources to improve student health. For example, adolescent mental health and suicide risk remain substantial public health concerns. Identifying protective factors that could foster positive mental health is a critical need. One report found that 39.7% of students experienced persistent sadness and hopelessness, 28.5% experienced poor mental health, 20.4% seriously considered attempting suicide, and 9.5% had attempted suicide. Findings indicate that protective factors (e.g., physical activity, having a household adult that always tried to meet a student’s basic needs, and school connectedness) are associated with lower prevalence of poor mental health and suicide risk (*18*). In another area of interest, school administrators are looking to recover learning losses and narrow academic disparities that widened during the COVID-19 pandemic. Students’ regular breakfast consumption might help reinforce these efforts. Nationally, 17.9% of students skipped breakfast every day. Skipping breakfast was positively associated with feeling persistently sad or hopeless and negatively associated with school connectedness and getting mostly As and Bs in school (*19*). Findings from the report on physical activity indicated that having a negative safety experience at school often was associated with a higher prevalence of meeting a physical activity guideline. For example, among female students, those who were threatened or injured with a weapon at school were more likely to meet the aerobic guideline of exercising ≥60 minutes/day 7 days a week. Conversely, negative safety experiences at school were associated with a lower prevalence of attending a physical education class. Understanding physical activity behaviors in the context of negative safety experiences is important because only 50% of students meet physical activity guidelines and attend a physical education class on all 5 school days (*20*).

## Limitations

Each report in this supplement includes a limitations section pertaining to that specific report. In general, YRBSS findings are subject to at least six limitations. First, YRBSS data apply only to students in grades 9–12 who attend public and private schools in the United States. Homeschooled students are not included nor are persons who do not attend school; therefore, data are not representative of all persons in this age group. In 2022, approximately 5% of youths aged 14–17 years were not enrolled in school (https://nces.ed.gov/programs/digest/d23/tables/dt23_103.20.asp). Second, although the national sample is designed to provide nationally representative estimates, and weighting and nonresponse bias analyses yielded a sample generalizable to U.S. high school students, schools with lower per-pupil expenditure were less likely to participate (ICF, unpublished data, 2024). Third, the extent of underreporting or overreporting of health behaviors, experiences, and conditions cannot be determined, although most questions demonstrate substantial test-retest reliability (*1*). Fourth, students in schools in both the national sample and a site-specific sample were only surveyed once, often using the site-specific questionnaire rather than the national questionnaire. Consequently, not all students in the national sample were asked all questions; therefore, the total number of students answering each question varied. From the data, it is not possible to determine whether a response is missing because the question did not appear in that student’s questionnaire, the student did not answer the question, or the response was set to missing because of an out-of-range response or logical inconsistency. Fifth, YRBS data analyses are based on cross-sectional surveys and can only indicate association between variables, not causality. Finally, the survey is descriptive and not designed to explain the reasons behind any observed results.

## Conclusion

Despite its limitations, YRBSS remains the best source for quality data at the national, state, tribal, territorial, and local school district levels for monitoring health behaviors, experiences, and conditions that contribute to the leading causes of mortality and morbidity among U.S. high school students and that can lead to health problems as adults. Since its inception in 1991, YRBSS has collected data from approximately 5 million high school students in approximately 2,300 separate surveys. In 2023, in addition to the national data, 36 states, three tribal governments, five territories, and 21 local school districts received data representative of their high school student populations (Figure 1).

This overview report describes YRBSS methods for guiding the analyses presented in this *MMWR* supplement. A full description of 2023 YRBS results and downloadable data from the national and site-specific surveys are available (https://www.cdc.gov/yrbs/index.html).

## Footnotes

* 45 C.F.R. part 46.114; 21 C.F.R. part 56.114.
